# Crystal structure of the pharmaceutical impurity 2-(4-chloro­benzo­yl)pyridine

**DOI:** 10.1107/S2056989026009175

**Published:** 2026-09-08

**Authors:** Karan J. Singh, Joseph M. Tanski

**Affiliations:** a Department of Chemistry Vassar College,Poughkeepsie NY 12604 USA; University of Aberdeen, United Kingdom

**Keywords:** crystal structure, π-stacking, pharmaceutical impurity, benzo­phenone derivative

## Abstract

The title compound, C_12_H_8_ClNO, is a pharmaceutical impurity and possible inter­mediate in the synthesis of carbinoxamine. The aromatic rings of the mol­ecule twist relative to one another resulting in an inter­planar angle of 38.1 (1)°. In the extended structure, the mol­ecules are packed into rippled sheets with offset face-to-face stacking of each aromatic ring with the equivalent ring in the neighboring mol­ecule.

## Abstract 2

The title compound is a pharmaceutical impurity and possible inter­mediate in the synthesis of carbinoxamine. The aromatic rings of the mol­ecule twist relative to one another resulting in an inter­planar angle of 38.1 (1)°. In the extended structure, the mol­ecules are packed into rippled sheets with offset face-to-face stacking of each aromatic ring with the equivalent ring in the neighboring mol­ecule.

## 1. Chemical context

2-(4-Chloro­benzo­yl)pyridine, C_12_H_8_ClNO (**I**), also known as carbinoxamine related compound A, is a known manufacturing impurity of the pharmaceutical ingredient carbinoxamine, 2-[(4-chloro­phen­yl)-pyridin-2-yl-meth­oxy]-*N*,*N*-dimethyl-ethanamine (C_16_H_19_ClN_2_O). The compound may be used to synthesize enanti­opure alcohols, such as (*S*)-(4-chloro­phen­yl)(pyridin-2-yl)methanol, as precursors for the synthesis of carbinoxamine in the fungus *Geotrichum candidum* using a mutated aceto­phenone reductase enzyme that catalyzes the reduction of ketones to alcohols (Tang *et al.*, 2024 ▸). This enzyme belongs to the alcohol de­hydrogenase (ADH) class, which catalyzes the reversible inter­conversion between ketones and alcohols. Carbinoxamine maleate, the maleic acid salt of carbinoxamine, is a first-generation H_1_-anti­histamine used to treat allergy symptoms and may be synthesized *via* a Grignard reaction: *para*-chloro­phenyl­magnesium bromide, formed by the addition of *para*-bromo­chloro­benzene to magnesium in anhydrous ether, is treated with 2-pyridine­aldehyde and subsequently with sodium and 2-di­methyl­amino­ethyl chloride to produce an approximate 65% yield of the medication (Mansukhbhai & Das, 2021 ▸). Carbinoxamine maleate performs by binding to inactive H_1_ receptors belonging to G-protein-coupled receptors (GPCRs) found in cells and stabilizing them to remain inactive. This mechanism prevents the binding of the chemical signal histamine to the receptors and inhibits signal transduction pathways leading to allergic reactions (Leurs *et al.*, 2002 ▸). As part of our studies in this area, we now describe the crystal structure of (**I**).
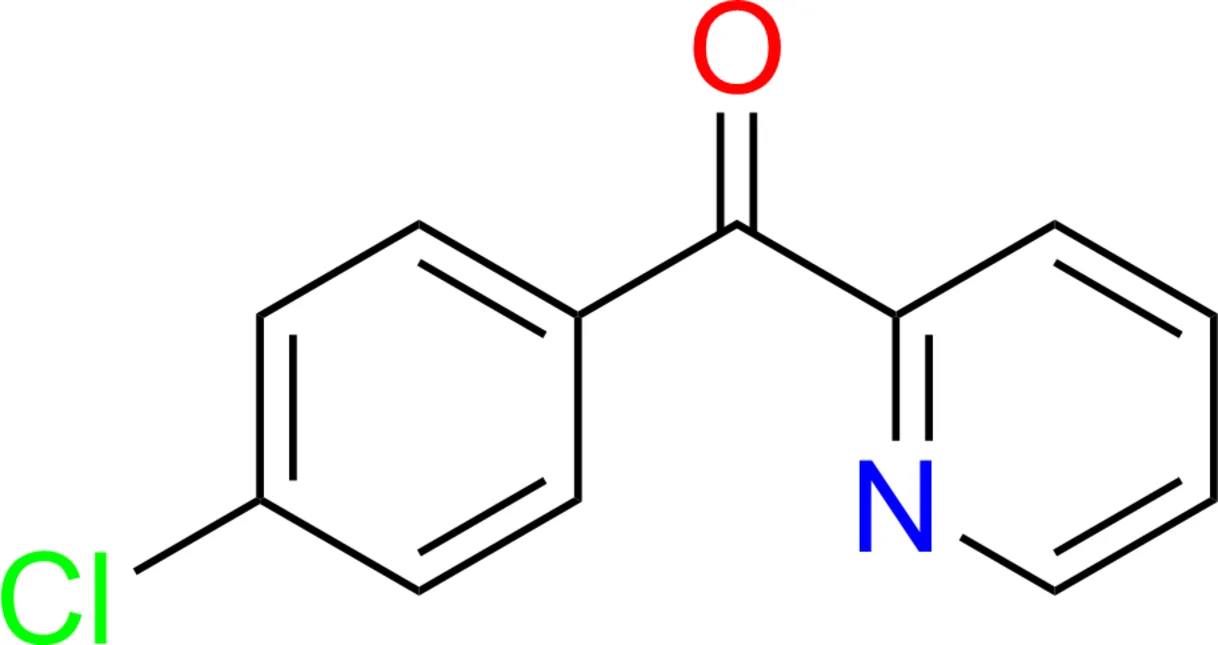


## 2. Structural commentary

**Figure 1:**
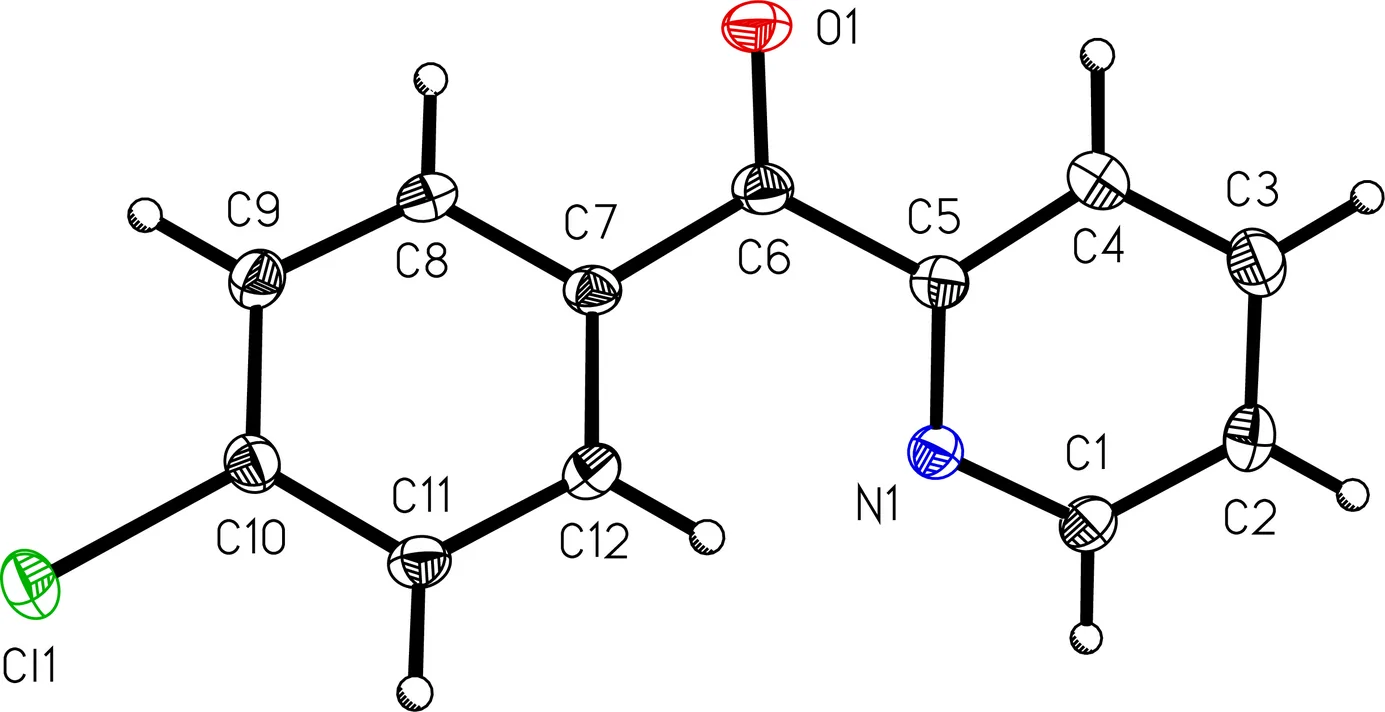
The mol­ecular structure of (**I**) with displacement ellipsoids shown at the 50% probability level.

Compound (**I**) crystallizes in space group *P*2_1_/*c* with one mol­ecule in the asymmetric unit. The mol­ecular structure of (**I**) (Fig. 1 ▸) reveals a 4-chloro­benzoyl group, consisting of a benzene ring bearing a chlorine-atom substituent at the *para* position relative to the carbonyl group, which is attached to the carbon atom *meta* to the nitro­gen atom of the pyridine ring. Individual mol­ecules of (**I**) adopt a twisted conformation that minimizes intra­molecular repulsion between the benzene and pyridine rings with an inter­planar angle of 38.1 (1)° between the C1–C5/N1 and C7–C12 rings. The C5—C6 [1.506 (5) Å] and C6—C7 [1.506 (5) Å] bond lengths are almost identical and the N1—C5—C6—O1 torsion angle is −152.6 (4)°.

## 3. Supra­molecular features

**Figure 2:**
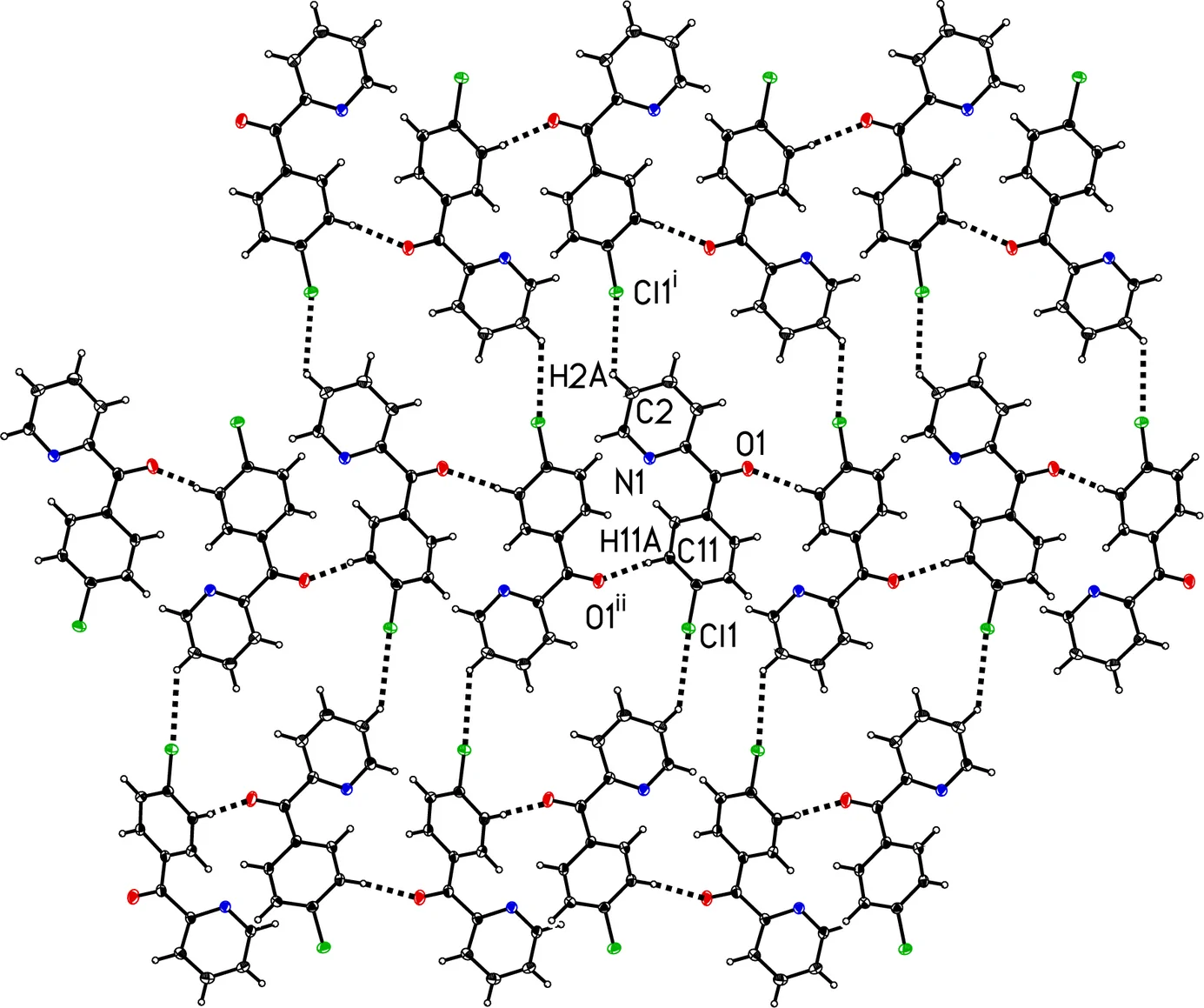
A view of the weak hydrogen-bonded sheets in (**I**). Displacement ellipsoids are shown at the 50% probability level. Symmetry codes: (i) *x* + 1, −*y* + 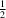, *z -* 1/2; (ii) −*x* + 1, *y* − 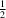, −*z* + 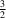.

**Figure 3:**
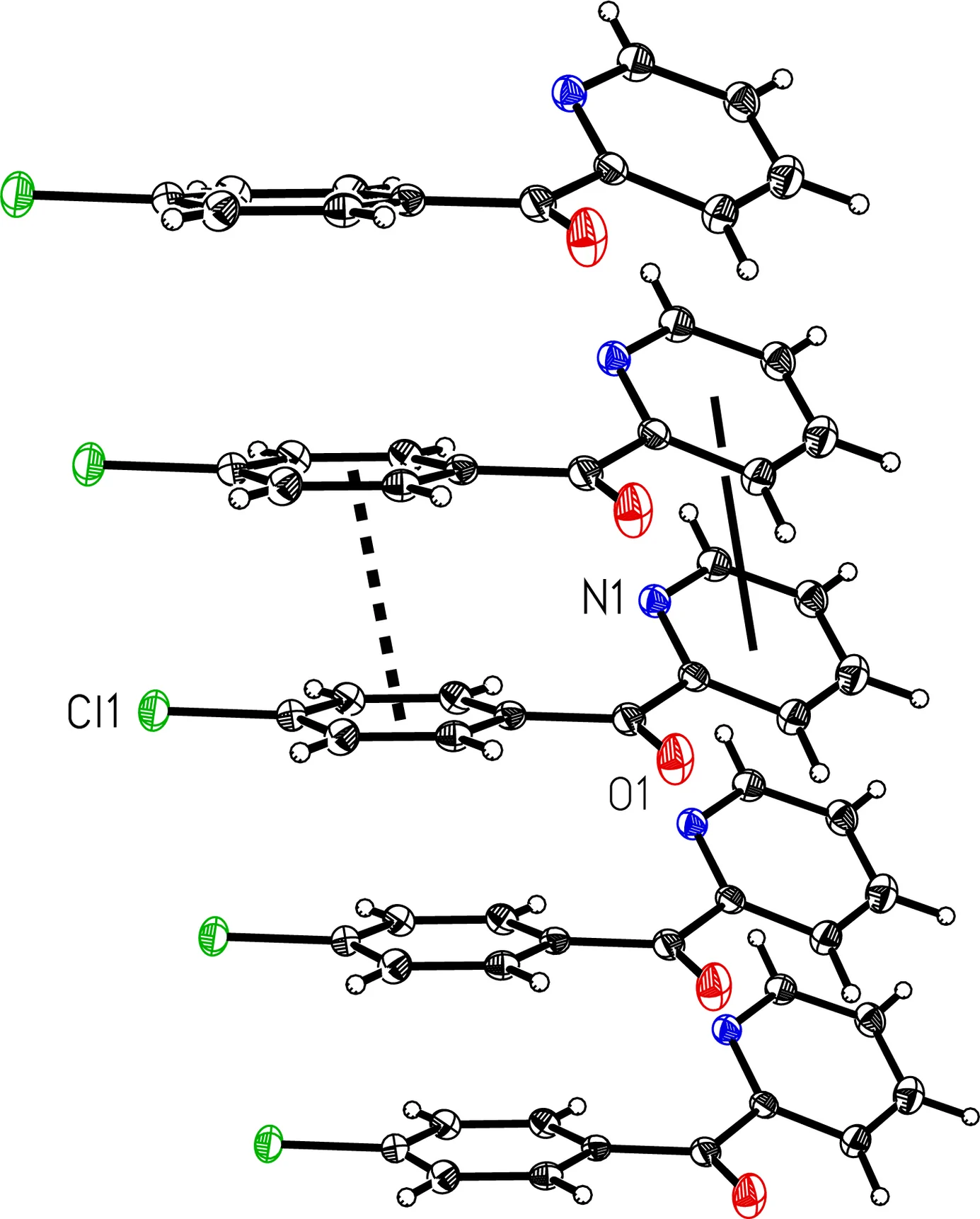
A view of the face-to-face π-stacking geometrical arrangement in (**I**) with a dashed line indicating the chloro­benzoyl inter­action and a solid line indicating the pyridine inter­action.

**Figure 4:**
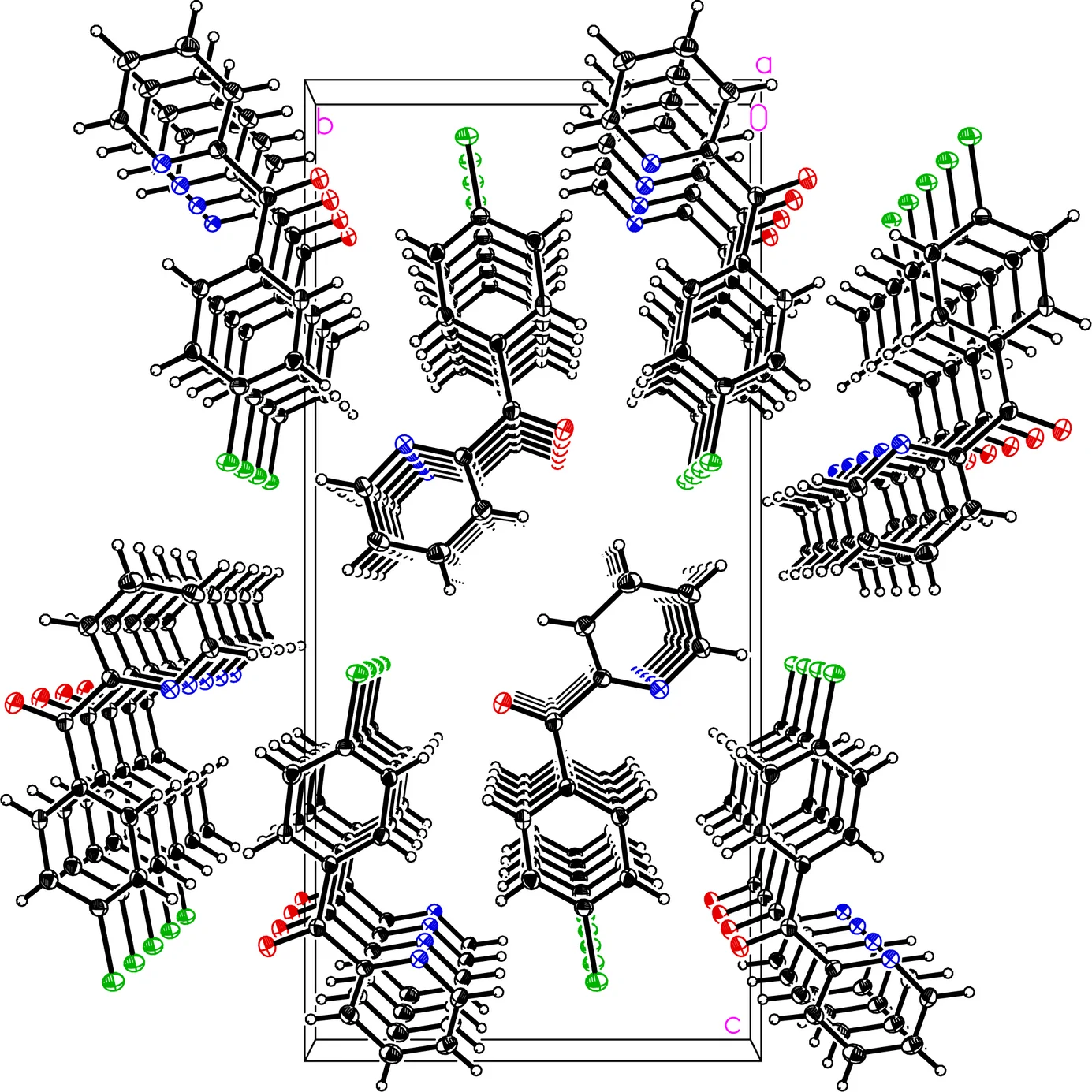
The packing in (**I**) viewed down [100].

In the extended structure, mol­ecules of (**I**) assemble into rippled (100) sheets *via* weak C—H⋯O and C—H⋯Cl inter­actions (Fig. 2 ▸, Table 1 ▸). As expected, the C11—H11*A*⋯O1 inter­action with a donor–acceptor distance of 3.406 (5) Å is shorter than the C2—H2*A*⋯Cl1 inter­action with a corresponding distance of 3.633 (4) Å. The shortest Cl⋯Cl contact of 3.894 (6) Å is much longer than the sum of the van der Waals radii for chlorine (3.50 Å; Bondi, 1964 ▸). The sheets stack in such a manner that a slipped face-to-face π-stacking geometrical arrangement of the rings is present between equivalent rings within the mol­ecules (Fig. 3 ▸). The π-stacking of the chloro­benzoyl rings is characterized by a centroid–centroid distance of 3.8943 (6) Å (the *a* unit-cell dimension) and a plane-to-centroid separation of 3.402 (3) Å, resulting in a ring shift of 1.895 (6) Å. The very similar π-stacking of the pyridine rings is characterized by the same centroid–centroid separation and a plane-to-centroid separation of 3.419 (3) Å, resulting in a ring shift of 1.865 (6) Å. In the overall packing (Fig. 4 ▸) there is also a longer C—H⋯N inter­action (not shown) that inter­connects the π-stacked sheets formed by the C—H⋯O and C—H⋯Cl inter­actions, with a C9—H*9A*⋯N1 donor–acceptor distance of 3.686 (5) Å.

## 4. Database survey

One similar compound to (**I**) was found in the CSD version 6.00, April 2025.; Groom *et al.*, 2016 ▸) with the CSD refcode CEYWOS (Syed *et al.*, 1984 ▸), which consists of a 4-chloro­benzoyl group attached to a pyridine ring. However, in contrast to (**I**), the 4-chloro­benzoyl group in CEYWOS is attached to the carbon atom *para* to the nitro­gen atom of the pyridine ring instead of the *meta* carbon atom. Both CEYWOS (space group *P*2_1_) and (**I**) display similar weak inter­molecular hydrogen bonding as well as π–π stacking inter­actions between the benzene and pyridine rings in parallel mol­ecules. However, CEYWOS exhibits a greater twist angle of the benzoyl and pyridine rings relative to one another, resulting in an inter­planar angle of 52.41° and a shortest Cl⋯Cl contact distance of 3.630 Å that is much closer to the sum of the van der Waals radii for chlorine.

## 5. Synthesis and crystallization

2-(4-Chloro­benzo­yl)pyridine (95%) was purchased from Enamine, USA, and recrystallized by slow evaporation over 48 h at room temperature using a 1:1 solvent mixture of ethyl acetate/hexane.

## 6. Refinement

Crystal data, data collection and structure refinement details are summarized in Table 2 ▸. Hydrogen atoms were included in calculated positions and refined using a riding model with C—H = 0.95 Å and *U*_iso_(H) = 1.2*U*_eq_(C).

## Supplementary Material

Crystal structure: contains datablock(s) global, I. DOI: 10.1107/S2056989026009175/hb8252sup1.cif

Structure factors: contains datablock(s) I. DOI: 10.1107/S2056989026009175/hb8252Isup2.hkl

Supporting information file. DOI: 10.1107/S2056989026009175/hb8252Isup3.cml

CCDC reference: 2584887

Additional supporting information:  crystallographic information; 3D view; checkCIF report

## Figures and Tables

**Table 1:** Hydrogen-bond geometry (Å, °)

*D*—H⋯*A*	*D*—H	H⋯*A*	*D*⋯*A*	*D*—H⋯*A*
C2—H2*A*⋯Cl1^i^	0.95	2.96	3.633 (4)	129
C11—H11*A*⋯O1^ii^	0.95	2.46	3.406 (5)	173

Symmetry codes: (i) 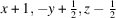; (ii) 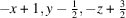.

**Table 2:** Experimental details

Crystal data	
Chemical formula	C_12_H_8_ClNO
*M*_r_	217.64
Crystal system, space group	Monoclinic, *P*2_1_/*c*
Temperature (K)	125
*a*, *b*, *c* (Å)	3.8943 (6), 10.8402 (15), 23.307 (3)
β (°)	90.573 (2)
*V* (Å^3^)	983.9 (2)
*Z*	4
Radiation type	Mo *K*α
μ (mm^−1^)	0.36
Crystal size (mm)	0.30 × 0.17 × 0.04

Data collection	
Diffractometer	Bruker APEXII CCD
Absorption correction	Multi-scan (*SADABS*; Krause *et al.*, 2015 ▸)
*T*_min_, *T*_max_	0.87, 0.99
No. of measured, independent and observed [*I* > 2σ(*I*)] reflections	22981, 3018, 2627
*R*_int_	0.047
(sin θ/λ)_max_ (Å^−1^)	0.715

Refinement	
*R*[*F*^2^ > 2σ(*F*^2^)], *wR*(*F*^2^), *S*	0.087, 0.234, 1.19
No. of reflections	3018
No. of parameters	136
H-atom treatment	H-atom parameters constrained
Δρ_max_, Δρ_min_ (e Å^−3^)	0.63, −0.92

Computer programs: *SAINT* and *SPEX3* (Bruker, 2013 ▸), *SHELXT2018/2* (Sheldrick, 2015*a* ▸), *SHELXL2017/1* (Sheldrick, 2015*b* ▸), *SHELXTL2014* (Sheldrick, 2008 ▸), *OLEX2* (Dolomanov *et al.*, 2009 ▸) and *Mercury* (Macrae *et al.*, 2020 ▸).

## supplementary crystallographic information

### 2-[(4-Chlorophenyl)carbonyl]pyridine Crystal data

**Table:**

C_12_H_8_ClNO	*F*(000) = 448
*M**_r_* = 217.64	*D*_x_ = 1.469 Mg m^−^^3^
Monoclinic, *P*2_1_/*c*	Mo *K*α radiation, λ = 0.71073 Å
*a* = 3.8943 (6) Å	Cell parameters from 9976 reflections
*b* = 10.8402 (15) Å	θ = 2.6–30.5°
*c* = 23.307 (3) Å	µ = 0.36 mm^−^^1^
β = 90.573 (2)°	*T* = 125 K
*V* = 983.9 (2) Å^3^	Plate, colourless
*Z* = 4	0.30 × 0.17 × 0.04 mm

### 2-[(4-Chlorophenyl)carbonyl]pyridine Data collection

**Table:**

Bruker APEXII CCD diffractometer	3018 independent reflections
Radiation source: sealed X-ray tube, Bruker APEXII CCD	2627 reflections with *I* > 2σ(*I*)
Graphite monochromator	*R*_int_ = 0.047
Detector resolution: 8.3333 pixels mm^-1^	θ_max_ = 30.6°, θ_min_ = 2.1°
φ and ω scans	*h* = −5→5
Absorption correction: multi-scan (SADABS; Krause *et al.*, 2015)	*k* = −15→15
*T*_min_ = 0.87, *T*_max_ = 0.99	*l* = −33→33
22981 measured reflections	

### 2-[(4-Chlorophenyl)carbonyl]pyridine Refinement

**Table:**

Refinement on *F*^2^	Primary atom site location: dual
Least-squares matrix: full	Secondary atom site location: difference Fourier map
*R*[*F*^2^ > 2σ(*F*^2^)] = 0.087	Hydrogen site location: inferred from neighbouring sites
*wR*(*F*^2^) = 0.234	H-atom parameters constrained
*S* = 1.19	*w* = 1/[σ^2^(*F*_o_^2^) + (0.0373*P*)^2^ + 7.3622*P*] where *P* = (*F*_o_^2^ + 2*F*_c_^2^)/3
3018 reflections	(Δ/σ)_max_ < 0.001
136 parameters	Δρ_max_ = 0.63 e Å^−^^3^
0 restraints	Δρ_min_ = −0.92 e Å^−^^3^

### 2-[(4-Chlorophenyl)carbonyl]pyridine Special details

**Table:**

Geometry. All esds (except the esd in the dihedral angle between two l.s. planes) are estimated using the full covariance matrix. The cell esds are taken into account individually in the estimation of esds in distances, angles and torsion angles; correlations between esds in cell parameters are only used when they are defined by crystal symmetry. An approximate (isotropic) treatment of cell esds is used for estimating esds involving l.s. planes.

### 2-[(4-Chlorophenyl)carbonyl]pyridine Fractional atomic coordinates and isotropic or equivalent isotropic displacement parameters (Å^2^)

**Table:**

	*x*	*y*	*z*	*U*_iso_*/*U*_eq_	
Cl1	0.1218 (3)	0.36829 (10)	0.89790 (4)	0.0240 (3)	
O1	0.3127 (10)	0.5625 (3)	0.62865 (14)	0.0306 (8)	
N1	0.3630 (9)	0.2415 (3)	0.61545 (14)	0.0182 (6)	
C1	0.4401 (11)	0.1573 (4)	0.57571 (17)	0.0209 (8)	
H1B	0.375692	0.074018	0.582413	0.025*	
C2	0.6100 (11)	0.1851 (4)	0.52497 (18)	0.0220 (8)	
H2A	0.66341	0.121867	0.498278	0.026*	
C3	0.6995 (11)	0.3065 (4)	0.51413 (19)	0.0247 (8)	
H3A	0.810253	0.328332	0.47945	0.03*	
C4	0.6246 (11)	0.3954 (4)	0.55481 (17)	0.0209 (8)	
H4A	0.687546	0.479185	0.549092	0.025*	
C5	0.4537 (10)	0.3588 (4)	0.60456 (16)	0.0167 (7)	
C6	0.3522 (11)	0.4574 (4)	0.64667 (17)	0.0186 (7)	
C7	0.3001 (10)	0.4297 (3)	0.70928 (16)	0.0159 (7)	
C8	0.1338 (10)	0.5204 (3)	0.74168 (17)	0.0165 (7)	
H8A	0.055346	0.593758	0.723501	0.02*	
C9	0.0826 (10)	0.5041 (4)	0.79999 (17)	0.0193 (7)	
H9A	−0.027204	0.566012	0.82209	0.023*	
C10	0.1959 (10)	0.3951 (4)	0.82540 (16)	0.0172 (7)	
C11	0.3722 (10)	0.3054 (4)	0.79449 (17)	0.0174 (7)	
H11A	0.458215	0.233698	0.813115	0.021*	
C12	0.4203 (10)	0.3227 (4)	0.73593 (17)	0.0174 (7)	
H12A	0.534994	0.261473	0.714114	0.021*	

### 2-[(4-Chlorophenyl)carbonyl]pyridine Atomic displacement parameters (Å^2^)

**Table:**

	*U*^11^	*U*^22^	*U*^33^	*U*^12^	*U*^13^	*U*^23^
Cl1	0.0307 (5)	0.0248 (5)	0.0165 (4)	−0.0023 (4)	0.0050 (3)	0.0009 (4)
O1	0.054 (2)	0.0156 (14)	0.0218 (15)	0.0020 (14)	0.0022 (14)	0.0033 (12)
N1	0.0213 (16)	0.0166 (15)	0.0168 (15)	−0.0006 (12)	−0.0001 (12)	−0.0006 (12)
C1	0.0264 (19)	0.0161 (17)	0.0201 (18)	−0.0016 (15)	0.0015 (15)	−0.0030 (14)
C2	0.0225 (19)	0.025 (2)	0.0184 (18)	0.0022 (16)	0.0024 (14)	−0.0043 (15)
C3	0.024 (2)	0.029 (2)	0.0208 (19)	0.0014 (17)	0.0040 (15)	0.0023 (16)
C4	0.0223 (18)	0.0207 (19)	0.0198 (18)	−0.0013 (15)	0.0011 (14)	0.0029 (15)
C5	0.0184 (16)	0.0159 (16)	0.0156 (16)	−0.0017 (13)	−0.0031 (13)	−0.0002 (13)
C6	0.0233 (18)	0.0147 (16)	0.0176 (17)	−0.0021 (14)	−0.0022 (14)	0.0005 (13)
C7	0.0181 (16)	0.0132 (16)	0.0163 (16)	−0.0027 (13)	−0.0016 (13)	0.0002 (13)
C8	0.0163 (16)	0.0123 (16)	0.0210 (17)	−0.0001 (13)	−0.0007 (13)	−0.0003 (13)
C9	0.0200 (18)	0.0177 (17)	0.0204 (18)	0.0012 (14)	0.0038 (14)	−0.0018 (14)
C10	0.0190 (17)	0.0178 (17)	0.0149 (16)	−0.0024 (13)	0.0004 (13)	−0.0001 (13)
C11	0.0198 (17)	0.0142 (16)	0.0181 (17)	0.0015 (13)	−0.0030 (13)	0.0008 (13)
C12	0.0189 (17)	0.0144 (17)	0.0188 (17)	0.0014 (13)	−0.0004 (13)	−0.0022 (13)

### 2-[(4-Chlorophenyl)carbonyl]pyridine Geometric parameters (Å, º)

**Table:**

Cl1—C10	1.741 (4)	C5—C6	1.506 (5)
O1—C6	1.224 (5)	C6—C7	1.506 (5)
N1—C1	1.337 (5)	C7—C12	1.394 (5)
N1—C5	1.345 (5)	C7—C8	1.403 (5)
C1—C2	1.394 (6)	C8—C9	1.387 (5)
C1—H1B	0.95	C8—H8A	0.95
C2—C3	1.385 (6)	C9—C10	1.392 (6)
C2—H2A	0.95	C9—H9A	0.95
C3—C4	1.386 (6)	C10—C11	1.395 (5)
C3—H3A	0.95	C11—C12	1.392 (5)
C4—C5	1.400 (5)	C11—H11A	0.95
C4—H4A	0.95	C12—H12A	0.95
			
C1—N1—C5	117.0 (3)	C12—C7—C8	119.9 (3)
N1—C1—C2	123.5 (4)	C12—C7—C6	123.3 (3)
N1—C1—H1B	118.3	C8—C7—C6	116.7 (3)
C2—C1—H1B	118.3	C9—C8—C7	120.7 (4)
C3—C2—C1	118.9 (4)	C9—C8—H8A	119.7
C3—C2—H2A	120.6	C7—C8—H8A	119.7
C1—C2—H2A	120.6	C8—C9—C10	118.5 (4)
C2—C3—C4	118.8 (4)	C8—C9—H9A	120.8
C2—C3—H3A	120.6	C10—C9—H9A	120.8
C4—C3—H3A	120.6	C9—C10—C11	121.8 (4)
C3—C4—C5	118.3 (4)	C9—C10—Cl1	120.0 (3)
C3—C4—H4A	120.8	C11—C10—Cl1	118.2 (3)
C5—C4—H4A	120.8	C12—C11—C10	119.0 (4)
N1—C5—C4	123.5 (4)	C12—C11—H11A	120.5
N1—C5—C6	118.5 (3)	C10—C11—H11A	120.5
C4—C5—C6	117.9 (4)	C11—C12—C7	120.0 (4)
O1—C6—C7	120.0 (4)	C11—C12—H12A	120.0
O1—C6—C5	118.0 (4)	C7—C12—H12A	120.0
C7—C6—C5	121.9 (3)		
			
C5—N1—C1—C2	0.7 (6)	C5—C6—C7—C12	16.5 (6)
N1—C1—C2—C3	−1.2 (7)	O1—C6—C7—C8	13.9 (6)
C1—C2—C3—C4	1.6 (7)	C5—C6—C7—C8	−166.2 (4)
C2—C3—C4—C5	−1.5 (6)	C12—C7—C8—C9	−1.0 (6)
C1—N1—C5—C4	−0.7 (6)	C6—C7—C8—C9	−178.4 (4)
C1—N1—C5—C6	176.4 (4)	C7—C8—C9—C10	−0.9 (6)
C3—C4—C5—N1	1.1 (6)	C8—C9—C10—C11	3.1 (6)
C3—C4—C5—C6	−176.0 (4)	C8—C9—C10—Cl1	−177.5 (3)
N1—C5—C6—O1	−152.6 (4)	C9—C10—C11—C12	−3.4 (6)
C4—C5—C6—O1	24.6 (6)	Cl1—C10—C11—C12	177.1 (3)
N1—C5—C6—C7	27.5 (6)	C10—C11—C12—C7	1.5 (6)
C4—C5—C6—C7	−155.3 (4)	C8—C7—C12—C11	0.6 (6)
O1—C6—C7—C12	−163.4 (4)	C6—C7—C12—C11	177.8 (4)

### 2-[(4-Chlorophenyl)carbonyl]pyridine Hydrogen-bond geometry (Å, º)

**Table:**

*D*—H···*A*	*D*—H	H···*A*	*D*···*A*	*D*—H···*A*
C2—H2*A*···Cl1^i^	0.95	2.96	3.633 (4)	129
C11—H11*A*···O1^ii^	0.95	2.46	3.406 (5)	173

Symmetry codes: (i) *x*+1, −*y*+1/2, *z*−1/2; (ii) −*x*+1, *y*−1/2, −*z*+3/2.
